# Predicting Nurse Turnover for Highly Imbalanced Data Using the Synthetic Minority Over-Sampling Technique and Machine Learning Algorithms

**DOI:** 10.3390/healthcare11243173

**Published:** 2023-12-15

**Authors:** Yuan Xu, Yongshin Park, Ju Dong Park, Bora Sun

**Affiliations:** 1 School of Maritime Economics and Management, Collaborative Innovation Center for Transport Studies, Dalian Maritime University, 1 Linghai Road, Dalian 116026, China; yuan.xu@dlmu.edu.cn; 2 Department of Marketing, Operations, and Analytics, Bill Munday School of Business, St. Edward’s University, 3001 South Congress, Austin, TX 78704, USA; 3 Department of Maritime Police and Production System, Gyeongsang National University, Tongyeong-si 53064, Gyeongsangnam-do, Republic of Korea; 4 School of Nursing, The University of Texas Austin, 1710 Red River St., Austin, TX 78712, USA; borang77@utexas.edu

**Keywords:** nurse turnover, machine learning, SMOTE, NSSRN, random forest, XGBoost

## Abstract

Predicting nurse turnover is a growing challenge within the healthcare sector, profoundly impacting healthcare quality and the nursing profession. This study employs the Synthetic Minority Over-sampling Technique (SMOTE) to address class imbalance issues in the 2018 National Sample Survey of Registered Nurses dataset and predict nurse turnover using machine learning algorithms. Four machine learning algorithms, namely logistic regression, random forests, decision tree, and extreme gradient boosting, were applied to the SMOTE-enhanced dataset. The data were split into 80% training and 20% validation sets. Eighteen carefully selected variables from the database served as predictive features, and the machine learning model identified age, working hours, electric health record/electronic medical record, individual income, and job type as important features concerning nurse turnover. The study includes a performance comparison based on accuracy, precision, recall (sensitivity), F1-score, and AUC. In summary, the results demonstrate that SMOTE-enhanced random forests exhibit the most robust predictive power in the classical approach (with all 18 predictive variables) and an optimized approach (utilizing eight key predictive variables). Extreme gradient boosting, decision tree, and logistic regression follow in performance. Notably, age emerges as the most influential factor in nurse turnover, with working hours, electric health record/electronic medical record usability, individual income, and region also playing significant roles. This research offers valuable insights for healthcare researchers and stakeholders, aiding in selecting suitable machine learning algorithms for nurse turnover prediction.

## 1. Introduction

The healthcare sector in the United States has undergone a remarkable transformation over the past few decades. Not only has it expanded significantly, but it has also become a driving force behind the nation’s economic growth, employing approximately 14.3 million individuals. With projections indicating the creation of an additional 3.2 million healthcare-related jobs soon [1], the healthcare industry’s significance in the American economy is set to soar even higher. Beyond its economic impact, healthcare is pivotal in American citizens’ lives, as it is fundamentally dedicated to supporting their health and well-being. Healthcare competition has dramatically increased in recent years especially due to the COVID-19 pandemic [2]. Despite the sector’s overall commendable performance, significant challenges persist.

One of the most pressing issues plaguing the US healthcare system is the problem of high employee turnover, particularly among nurses. This turnover impacts the healthcare industry’s ability to deliver quality care and hampers its overall performance. Many nurses leave their current organizations in pursuit of opportunities to enhance their skills and competencies [3]. This phenomenon, called turnover intention, measures how much employees think about leaving their current organization. This significantly affects the organization’s sustainability and reputation [4]. Turnover intention represents a process wherein employees contemplate leaving their current organization for various reasons, reflecting their anticipation of voluntarily departing soon [2]. It underscores an employee’s contemplation and inclination toward seeking alternative employment. In the healthcare industry, nurse turnover intention has emerged as a pervasive problem, transcending organizational size, location, and nature of business [4]. The adverse impact of high turnover intention on healthcare organizations is keenly felt, as it directly affects the quality of service they can provide [5]. 

International studies consistently report a significant increase in nurses expressing their intention to leave their jobs [6,7]. Hence, the ability to predict nurse turnover has become a crucial procedure for healthcare organizations. Early access to information regarding nurse turnover status empowers organizations to take preemptive measures and implement interventions to curtail turnover, ultimately ensuring the continued delivery of high-quality healthcare services [8]. This study aimed to develop and evaluate a predictive model for nurse turnover in the United States (U.S) using machine learning. 

The remainder of this research paper is as follows. Section 2 presents a literature review. Section 3 presents methodology such as data preprocessing, the ML algorithm, and the SMOTE method. Section 4 presents the experimental results of the study and compares them with existing methods. Section 5 presents the study’s conclusion and future research.

## 2. Literature Review

Extensive research efforts have been dedicated to understanding and evaluating nurse turnover, specifically identifying predictive factors for nurse turnover intention [3]. Traditional approaches for determining predictive factors on nurse turnover have heavily relied on statistical approaches using regression and ANOVA analysis, which are commonly used tools in applied econometrics [7,9]. Nowadays, big data exists in the healthcare industry. Considering the fact that nurse turnover is influenced by numerous factors, traditional methods such as regression or ANOVA analysis are inadequate in fully capturing the complex relationships within turnover. Machine learning effectively extracts patterns and makes consistent decisions, especially in tasks associated with high-dimensional data [6]. 

Artificial intelligence (AI) uniquely analyzes diverse datasets, from structured human resource records to unstructured sources like social media sentiment and employee feedback [10]. This holistic approach provides valuable insights into the factors contributing to turnover. Such factors include work-related stress, job dissatisfaction, or personal circumstances [11]. Human resource departments can identify early warning signs such as increased absenteeism or declining performance of employees [12]. Thus, the healthcare industry can proactively intervene in the turnover intention based on predictive factors. These interventions may include tailored training programs, workload adjustments, or personalized support to address employee concerns [13]. 

One of the main branches of AI is machine learning (ML) algorithms, which can learn and adapt knowledge based on data training and learn from recurring patterns from the dataset. Then, observed data patterns are used to predict an outcome. Various machine learning algorithms were popular for predicting the outcomes in the recent healthcare-related studies [14,15], which included but were not limited to neural networks (NN), extreme gradient boosting (XGBoost), random forest (RF), decision tree (DT), logistic regression (LR), and support vector machine (SVM) [7,9,13]. In ML, classification algorithms consider that every class should have an approximately equal number, but, in practice, this may fail due to class imbalances [16]. In an imbalanced dataset, we have the class with fewer examples, a so-called minority class, and the class with many examples, a so-called majority class. If an imbalanced dataset is used when performing ML analysis, the imbalanced distribution of the classes may be overlooked. This results in poor performance for the minority class, creating a model bias for the majority class because ML tends to learn more about the majority class during the data partitioning process [17].

The academic significance of our present research lies in the scarcity of open literature studies focused on nurse turnover prediction using machine learning algorithms. While numerous papers have examined the association between various factors and nurse turnover, only a few have delved into the predictive potential of machine learning in this context. Demographic factors such as age, sex, marital status, work experience, and job position have commonly been identified as contributing factors to nurse turnover [18]. Organizational factors, including department, employment status (regular or non-regular), and lower nursing grade, have also been found to predict turnover [19]. Furthermore, research from South Korea highlights additional critical factors such as marriage, childbirth, and child-rearing as significant contributors to nurse turnover [7,19,20,21]. However, it is essential to note that the most recent study conducted by Bae (2023) employed the 2018 National Sample Survey of Registered Nurses (NSSRN) dataset and utilized multivariable logistic regression for analysis. One notable challenge encountered in the study was dealing with imbalanced data in the context of turnover classification. This challenge serves as a key motivation for our research. 

Previous literature reviews have demonstrated that existing approaches have effectively predicted nurse turnover across various datasets. However, diverse machine learning algorithms have been employed without considering class imbalance issues to enhance various performance metrics, including accuracy, precision, and recall. In this study, our primary objective is to compare machine learning techniques alongside the Synthetic Minority Over-sampling Technique (SMOTE) to determine the most effective method for predicting nurse turnover. This is the first endeavor to analyze all dataset features within the NSSRN context comprehensively.

## 3. Method

### 3.1. Research Framework for Nurse Turnover Prediction Model

**Figure 1:**
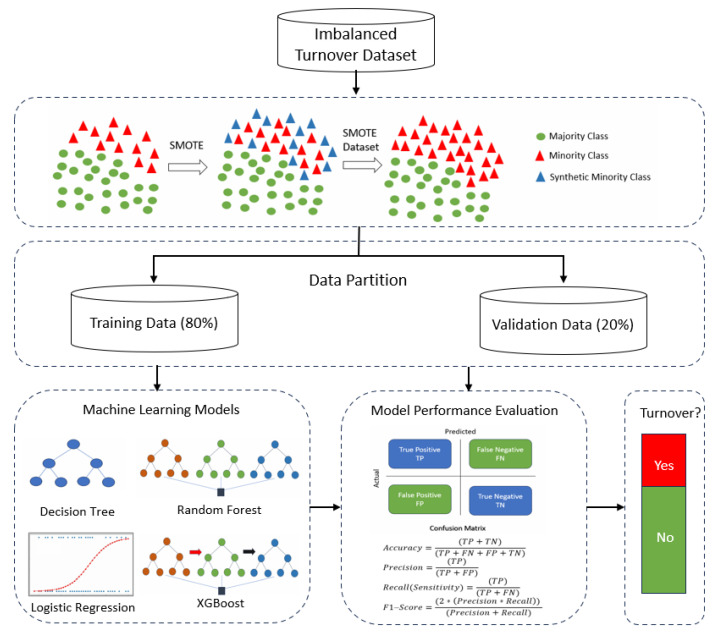
Overall Framework of Nurse Turnover Prediction.

First and foremost, data preprocessing was carried out. This phase involved handling missing values and creating dummy variables for categorical data. Once the data preprocessing was complete, the next phase involved the application of the SMOTE method. The objective was to rectify class imbalance in nurse turnover samples between the training (80%) and validation (20%) datasets. This step aimed to enhance the accuracy of the machine learning models used for nurse turnover prediction by increasing the sample size. The SMOTE, an oversampling technique, was chosen for this task due to its effectiveness in addressing the issue of highly imbalanced data, a common challenge in machine learning studies. The SMOTE is known as the most dominant technique that can be used to address class imbalance by generating random synthetic data from minority classes by nearest neighbors using Euclidean distance. Therefore, new instances become very similar to the original dataset because new instances are generated based on original features [22]. Following the resolving of data imbalance, the subsequent phase entailed the development of machine learning algorithms for training and predicting nurse turnover. Four distinct models were employed: LR, RF, DT, and XGBOOST. A grid search was used to select the best parameters for each model to optimize the performance of these models. Afterward, the performance of these models was assessed using five key performance metrics: accuracy, recall (sensitivity), precision, F1-score, and area under the curve (AUC). The overall framework of the proposed intelligent approach for predicting nurse turnover is visually represented in Figure 1.

### 3.2. Data Collection and Data Preprocessing 

We conducted a study using the publicly available 2018 NSSRN to estimate nurse turnover rates in the United States, as the Health Resources and Services Administration (HRSA) reported in 2023 [23]. The NSSRN is designed to capture various characteristics of nurses, including demographics, employment details, and licensing and certification status. Data were collected from April to October 2018, with 102,520 registered nurses (RNs) invited to participate. A total of 50,273 nurses completed the survey, resulting in an unweighted response rate of 50.1% and a weighted response rate of 49.1%. Out of the entire dataset, 13% of the values were missing across various variables, namely Electronic Health Record (EHR) or Electronic Medical Record (EMR), Employment_Type, Job_Type, Employment_Setting, Working_Hour, Practice, Individual_Income, and Job_Satisfaction. Due to the substantial number of missing values, precisely eight null values for each record, the decision was made to delete these instances from the dataset for data completeness and analysis accuracy.

Our study focused on RNs, Nurse Practitioners (NPs), Clinical Nurse Specialists (CNSs), Nurse Anesthetists (NAs), and Nurse-Midwives (NMs) who were working as of 31 December 2017. After excluding records with missing values, our dataset included 43,987 samples. Among these records, 89% indicated turnover (“Yes”), while 11% indicated no turnover (“No”), indicating an imbalanced dataset.

For our analysis, we selected 18 relevant variables from the NSSRN database based on the prior literature [2,6,7]. These variables are listed in Table 1, and we renamed them from the NSSRN codebook for clarity. We converted categorical variables into factor levels to facilitate machine learning analysis, as ML algorithms require numerical inputs [10]. Binary dummy variables for categorical variables were generated, and the number of dummy variables created corresponded to one less than the original number of categories in Table 1. Subsequently, we split the dataset into an 80% training set and a 20% validation set. 

### 3.3. Sampling Method

**Figure 2:**
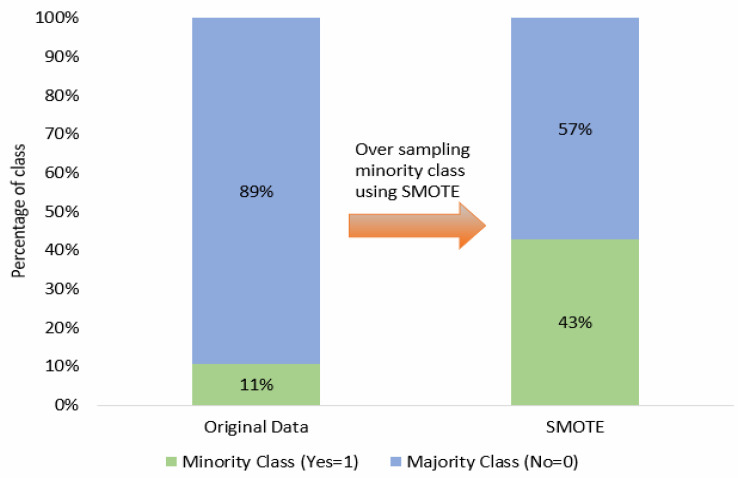
Illustration of SMOTE process for turnover data.

After establishing training and validation datasets, we employed the SMOTE to rectify the class imbalance issue within the new training dataset. This approach substantially improved the distribution of each class, mitigating any potential bias towards the minority class [24]. The SMOTE accomplished this by augmenting the quantity of data instances by generating synthetic data points for the minority class derived from its nearest neighbors based on the Euclidean distance metric [22]. As a result, the newly generated instances exhibited a heightened resemblance to the original data distribution [25]. Before applying the SMOTE, the class distribution for nurse turnover displayed a majority-minority split of 89% and 11%, respectively. However, following the implementation of the SMOTE method, these proportions shifted to 57% and 43%. A visual representation of the SMOTE’s impact on our turnover dataset can be observed in Figure 2.

**Figure 3:**
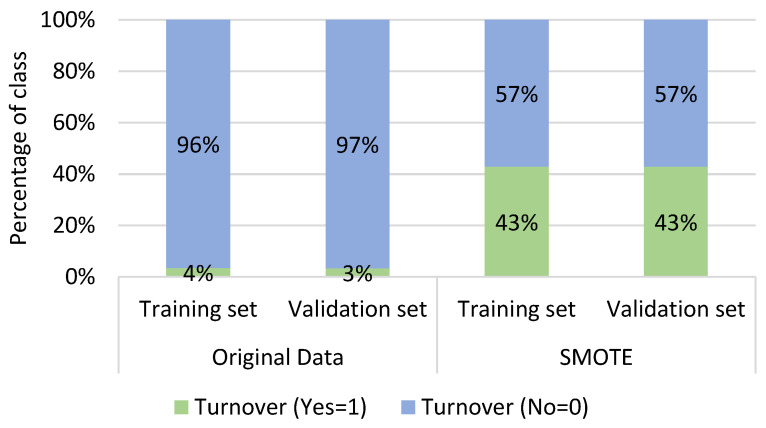
Distribution of the classes in the training and validation sets in the original and SMOTE dataset.

The distribution of classes in both the training and validation sets was illustrated in Figure 3 for both the original and SMOTE datasets. In the original dataset, there were notable variations in the turnover classes (Yes and No) within both the training and validation sets. However, following the application of the SMOTE, the classes exhibited a more balanced distribution. 

### 3.4. Machine Learning Models

#### 3.4.1. Decision Tree

DT is a non-parametric supervised learning algorithm for prediction and classification [26]. A decision tree-like structure contains internal, branch, and leaf nodes. Each internal node represents a judgment on an attribute, each branch represents the output of a judgment, and each leaf node represents a prediction or classification result. A decision tree is a root-to-leaf recursive process, including feature selection, construction, and pruning. 

Feature selection is selecting an appropriate attribute to partition the sample at each node. It is important as it can decide the decision tree’s breadth and depth. The goal is to make the classified dataset relatively pure, which means records resembling each other in each classified portion. The Gini index or Entropy measure can measure a dataset’s impurity. The Gini index is mainly used as a classification standard in the classification and regression tree (CART) decision tree algorithm. In this study, we use CART as a predictive algorithm, which is good at handling both continuous and discrete variables.

The formula of the Gini index for dataset A is shown in Equation (1). In the equation, k is one class of the dependent variable and $p_{k}$ is the proportion of records in a classified portion that belong to class k. Evidently, the smaller the number of $Gini\left(A\right)$, the higher the purity of dataset A.

(1) $$Gini\left(A\right)=1-\sum_{k=1}^{m}p_{k}^{2}$$

When dataset A is binary split on a certain value x based on attribute X into two subsets $A_{1}$ and $A_{2}$, the Gini index for the split dataset A is shown in Equation (2). For a specific attribute X, calculate the corresponding Gini index for each value x separately and select the smallest value as the optimal binary scheme obtained by attribute X.

(2) $$Gini_{X=x}\left(A\right)=\frac{\left|A_{1}\right|}{A}Gini\left(A_{1}\right)+\frac{\left|A_{2}\right|}{A}Gini\left(A_{2}\right)$$

Then, repeat the process for all the attributes, obtain all the optimal binary schemes, and select the smallest of them as the dataset’s optimal segmentation attribute. 

Decision tree construction depends on the feature selection process. The whole dataset A is the root node. After obtaining the optimal attribute and value that yields the purest dataset, the resulting split points become nodes on the decision tree. This recursive partitioning process continues until a full-grown tree is constructed.

The final process is pruning the full-grown tree to avoid overfitting. Overfitting is a phenomenon in which the error rate of the training sample decreases to 0. Still, the error rate of the validation or test sample is pretty high as it has a first downward and then upward trend with the number of splits. The key to pruning is to find the point at which the error rate of the validation sample is at a minimum. The CART algorithm uses a validation dataset to prune back the full-grown tree generated by the training dataset. It uses a cost complexity pruning strategy that designs an indicator to measure the complexity cost of a subtree and prunes by setting a threshold at this cost. The greater the cost, the greater the deviation caused by pruning, that is, the less it can be pruned. 

#### 3.4.2. Random Forest 

RF is a multi-tree ensemble learning approach that applies the concept of Bagging to improve the weak generalization ability of a single decision tree [27]. Bagging, or bootstrap aggregating, is an algorithm that randomly selects several subsets as training data, uses them to construct several models, and then takes the average or majority vote as the output results. RF is a stable and effective classifier that integrates many decision trees. The process of constructing a single decision tree is represented in the previous section. The training data used to construct a tree are generated by random sampling with replacement from the whole dataset, assuming 80% of the total records in this study. 

Then, with numerous different training datasets, we construct many decision trees that form a random forest as a whole. Choosing the optimal number of decision trees in an RF is important as it relates to the correlation and classification ability of any two trees in the RF. This parameter can be decided by calculating and comparing the out-of-bag error for different RF models. The smaller the out-of-bag error is, the better the RF model is. The out-of-bag error is the ratio of misclassified records to the total number of records.

The class decides the classification or prediction of the final RF model with the majority vote of decision trees. For example, suppose an RF model consists of 100 decision trees. In that case, we find that the voting result of 70 trees is 1 for a specific record and the voting result of the other 30 trees is 0, so the final classification is 1 for this record. RF is good at handling high-dimensional data as well as imbalanced datasets at a fast speed. In addition, it can provide relative importance for different variables for decision-makers.

#### 3.4.3. Logistic Regression 

LR is a generalized linear regression analysis model mainly used for binary classification [28]. For binary LR, the dependent variable only has two classes denoted as 1 and 0, and the independent variables can be numerical and categorical. Assuming that under the impact of the independent variables ($x_{1},x_{2},\ldots ,\;x_{q}$), the probability of the dependent variable (y) being “1” is p, and the likelihood of being “0” is 1−p. Then, the goal of LR is to investigate the relationship between the probability p and the independent variables, shown in Equation (3). Odds denote the ratio of probabilities of the dependent variable (y) being “1” and being “0”, as shown in Equation (4). By combining Equations (3) and (4), we obtain Equation (5).

(3) $$p=\frac{1}{1+e^{-\left(\beta_{0}+\beta_{1}x_{1}+\beta_{2}x_{2}+\ldots +\beta_{q}x_{q}\right)}}$$

(4) $$Odds=\frac{p}{1-p}=e^{\beta_{0}+\beta_{1}x_{1}+\beta_{2}x_{2}+\ldots +\beta_{q}x_{q}}$$

Finally, taking natural logarithms on both sides of Equation (4), we can obtain the LR model, as shown in Equation (5). In Equation (5), $ln\left(\frac{p}{1-p}\right)$ is called logit, and it has a linear relationship with independent variables. The coefficients ($\beta_{0},\;\beta_{1},\beta_{2},\ldots ,\;\beta_{q}$) in the model are estimated using the Maximum Likelihood Estimate algorithm. The LR model has high computational efficiency and can clearly explain the impact of different independent variables on the dependent variable by checking the odds ratio.

(5) $$ln\left(\frac{p}{1-p}\right)=ln\left(odds\right)=\beta_{0}+\beta_{1}x_{1}+\beta_{2}x_{2}+\ldots +\beta_{q}x_{q}$$

#### 3.4.4. Extreme Gradient Boosting 

XGBoost is a widely used machine learning algorithm based on a decision tree ensemble [29]. It introduces parallel computing and regularization terms based on the original gradient boosting decision tree (GBDT) algorithm, thereby improving the model’s performance and computational efficiency. XGboost consists of decision trees, which are called “weak learners”. But unlike RF, the decision trees that makeup XGBoost have a sequential order, and the generation of the latter decision tree is related to the previous decision tree’s prediction. XGBoost is an additive model whose predicted value is the sum of the predicted values of all individual decision trees.

#### 3.4.5. Performance Metrics

The confusion matrix is used to evaluate different machine learning algorithms’ prediction and classification performance. The confusion matrix is a commonly used metric for classification. It is a situation analysis table that summarizes the records in the dataset in the form of a matrix according to the two criteria of the real category and the predicted category [22]. As shown in Table 2, the matrix columns represent the true values, and the matrix rows represent the predicted values [26].

- True Positive (TP): Records of actual “Yes” for turnover are correctly identified as “Yes”.
- False Negative (FN): Records of actual “Yes” for turnover are incorrectly identified as “No”.
- False Positive (FP): Records of actual “No” for turnover are incorrectly identified as “Yes”.
- True Negative (TN): Records of actual “No” for turnover are correctly identified as “No”.

The confusion matrix provides essential performance metrics, including accuracy, recall (sensitivity), precision, and the F1-score. These metrics are crucial indicators for evaluating the model’s performance [13]. The area under the curve (AUC) score maximizes recall and specificity, falling within the range of [0, 1]. AUC scores between 0.5 and 0.6 are considered inadequate, scores between 0.6 and 0.7 are typical, scores between 0.7 and 0.8 are good, scores between 0.8 and 0.9 are very good, and scores above 0.9 are deemed excellent [29]. We calculate the performance metrics based on the following equations:

(6) $$\mathrm{Accuracy}=\frac{\left(\mathrm{TP}+\mathrm{TN}\right)}{\left(\mathrm{TP}+\mathrm{FN}+\mathrm{FP}+\mathrm{TN}\right)}\;$$

(7) $$\mathrm{Precision}=\frac{\left(\mathrm{TP}\right)}{\left(\mathrm{TP}+\mathrm{FP}\right)}$$

(8) $$\mathrm{Recall}\left(\mathrm{Sensitivity}\right)=\frac{\left(\mathrm{TP}\right)}{\left(\mathrm{TP}+\mathrm{FN}\right)}$$

(9) $$\mathrm{F1-Score}=\frac{\left(2\times \left(\mathrm{Precision}\times \mathrm{Recall}\right)\right)\;}{\left(\mathrm{Precision}+\mathrm{Recall}\right)}$$

## 4. Results

### 4.1. Experiment Setup

All data processing, sampling, and machine learning analyses were conducted using the R statistical software (2022.02.0+492 version), a freely available open-source tool.

### 4.2. Characteristics of the Participants

The characteristics of 43,937 nurses are summarized in Table 3. A total of 4728 nurses (11%) left their primary nursing positions. Among the turnover group, those holding NP and RN qualifications tended to leave their positions, accounting for 45.96% and 44.67%, respectively. Most nurses expressed satisfaction with their primary nursing positions, with 9.77% reporting dissatisfaction and 90.23% reporting satisfaction. On average, the age of the nurses was 55 ± 11 years, individual income averaged $70,856 ± 41,404, and they worked an average of 346 ± 14.4 h per week. In terms of race, 86.51% of nurses were White, and 91.10% were female among those in the turnover group. Furthermore, 75.04% of those who left their positions were married, and 93.97% of nurses reported no prior military service. Regarding household income, 21.49% of nurses earned less than $75,000, 43.46% of nurses earned between $75,000 and $150,000, and 35.05% are more than $150,001. When it came to their educational backgrounds, more than half (57%) held advanced degrees such as MSN and PhD/DNP/DN. Most nurses (82.38%) did not have dependents under the age of 6, and 90.08% were hired by organizations and working full-time (79.61%). Regarding employment settings, 34.01% of nurses worked in clinical/ambulatory settings, followed by hospitals (43.53%) and inpatient/other settings (22.46%). Finally, 78.79% of nurses reported that they could practice to the extent of their knowledge, education, and training. Table 3 also displays the distribution of characteristics following the application of the SMOTE. Once again, the application of the SMOTE has effectively addressed the imbalance in classification. The newly created dataset retains information from the original dataset, as there are no significant variations in the distribution.

### 4.3. Machine Learning Analysis Results

In this study, we comprehensively analyzed supervised machine learning classifiers after implementing the SMOTE on our dataset. Our primary goal was to evaluate the predictive accuracy and performance of five distinct machine learning algorithms, namely SMOTE-enhanced Logistic Regression (SMOTE_LR), SMOTE-enhanced Random Forest (SMOTE_RF), SMOTE-enhanced Decision Trees (SMOTE_DT), and SMOTE-enhanced XGBoost (SMOTE_XGB), in the context of predicting nurse turnover. 

Table 4 displays the outcomes of the logistic regression (LR) model, presenting odds ratios (ORs), 95% confidence intervals (CIs), and *p*-values at a 95% significance level, which shed light on the influence of each variable on nurse turnover. Notably, we treated NP as the reference category. Individuals falling under the category of Other (comprising NA and NM) are 1.592 times more likely to experience turnover than those in the NP group, assuming all other variables remain constant (CI: 1.42–1.78). Nurses residing in the South and West regions show a decreased likelihood of turnover (OR = 1.037, CI: 0.95–1.14). Additionally, nurses who make use of Electronic Health Records (EHR) or Electronic Medical Records (EMR) technology exhibit a reduced likelihood of turnover (OR = 0.567, CI: 0.52–0.62).

When considering Employee by Organization as the reference category, other types of employment (such as travel nurses and the self-employed) are associated with a substantial increase in the odds of turnover (OR = 2.525, CI: 2.29–2.78). Among different job types, part-time nurses have 1.446 times the odds of turnover compared to their full-time counterparts under constant conditions. Furthermore, nurses working in inpatient or other settings exhibit a moderately increased likelihood of turnover (OR = 1.248, CI: 1.15–1.35). Notably, individuals working standard work hours are less likely to experience turnover (OR = 0.732, CI: 0.69–0.78). Having fewer opportunities for job practice is associated with an increased likelihood of turnover. Male nurses, single individuals, and veterans are more likely to experience turnover. Concerning race, White individuals are less likely to turnover (OR = 0.538, CI: 0.50–0.58). A household income of more than $150,001 significantly increases turnover, as indicated by the model (*p* < 0.05). On the other hand, individuals with a BSN (OR = 0.726, CI: 0.66–0.80) and MSN (OR = 0.730, CI: 0.80) are less likely to turnover. Having dependents under 6 years old is linked to a moderately increased likelihood of turnover (OR = 1.357, CI: 1.25–1.47). Lastly, higher age and nurse income were linked decreased nurse turnover.

**Figure 4:**
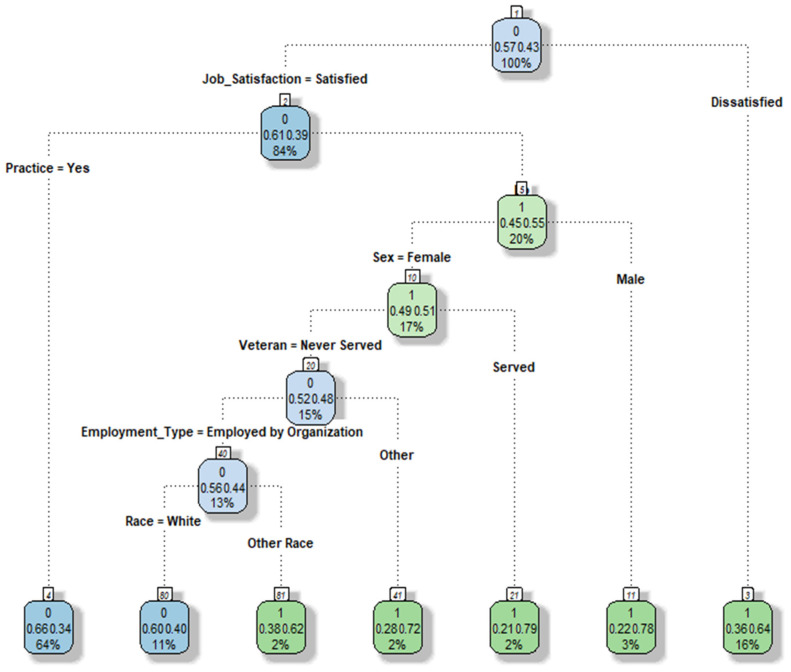
SMOTE_DT Results.

Figure 4 depicts the default decision tree analysis results for nurse turnover. At the root node (node 1), we find all the records from the training dataset, comprising 43% “Yes” and 57% “No” outcomes in our target variable (Turnover). The “0” within the top node’s box signifies the majority of nurses who did not leave their jobs.

The first node occurs at the Job Satisfaction node (node 2), where 84% of nurses report job satisfaction with a 39% turnover probability. In contrast, if nurses express dissatisfaction with their jobs (16%), they move to the terminal node (3) with a 64% probability of turnover.

Nurses who are satisfied with their jobs but cannot practice have a 55% chance of turnover (node 5). Notably, male nurses who could not practice in their jobs exhibited a higher turnover probability of 78%. Furthermore, nurses serving in the military, working as travel nurses, or in other roles, along with those of non-white ethnicity, show a notably high probability of turnover. The terminal nodes represent the final decision tree for nurse turnover. Among the seven terminal nodes, two are associated with the classification “Did not Turnover”, while four lead to the “Turnover” classification. The decision tree analysis identifies the most influential variables for turnover as Job Satisfaction, followed by Job Practice, Gender, Veteran status, Employee Type, and Race.

### 4.4. Feature Importance of ML Models

**Figure 5:**
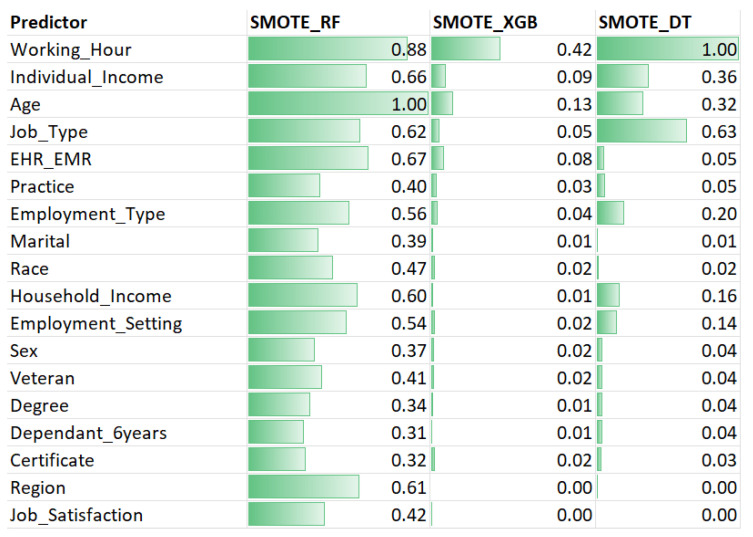
Feature Importance for predictor using SMOTE random forests, SMOTE_XGB, and SMOTE_DT.

Based on different feature importance criteria, SMOTE_RF, SMOTE_XGB, and SMOTE_DT provided importance rankings for relevant variables in predicting turnover using the mean decrease score. Figure 5 displays the mean decrease score and ranking of 18 variables under three different SMOTE-based ML models.

From SMOTE_RF, the top five important variables for predicting turnover were AGE (1), WORKING_HOUR (0.88), HER_EMR (0.67), INDIVIDUAL_INCOME (0.66), and JOB_TYPE (0.62). In the SMOTE_XGB results, WORKING_HOUR (0.42), AGE (0.13), INDIVIDUAL_INCOME (0.09), EHR_EMR (0.08), and JOB_TYPE (0.05) were identified as important features. SMOTE_DT revealed that WORKING_HOUR (1), JOB_TYPE (0.63), INDIVIDUAL_INCOME (0.36), AGE (0.32), and EMPLOYMENT_TYPE (0.02) were the most important features. Blytt et al. (2022) showed an association between working hours and turnover intention. Nurses with higher working hours tend to seek jobs with preferable working time arrangements. Age was an important factor in turnover intention. Previous research found that new graduate nurses, who are usually young, have a higher turnover than experienced nurses because they tend to quit their jobs to seek career advancement [20]. 

Conversely, SMOTE_RF, DEPENDANT_6YEARS (0.31), CERTIFICATE (0.32), DEGREE (0.34), SEX (0.37), and MARITAL (0.39) exhibited the lowest mean decrease scores, indicating that they are the least important variables for predicting nurse turnover. SMOTE_XGB, JOB_SATISFACTION (0), HOUSEHOLD_INCOME (0.01), MARITAL (0.01), DEGREE (0.01), and DEPENDANT_6YEARS (0.01) had the lowest mean decrease scores, making them the least important variables for prediction. SMOTE_DT identified REGION (0), JOB_SATISFACTION (0), MARITAL (0.01), RACE (0.02), and CERTIFICATE (0.03) as the least important variables. SMOTE_LR was excluded from the analysis because it provides variable importance for the entire set of predictive variables, preventing us from comparing variable rankings and their correlations. However, we compare SMOTE_LR with other models in terms of performance index.

We performed Pearson correlation analysis using mean decrease scores to determine if the important feature coincides with a similar pattern among different ML models. Equation (10) calculates the correlation coefficient.

(10) $$r=\frac{\sum \left(x_{i}-\bar{x}\right)\left(y_{i}-\bar{y}\right)}{\sqrt{\sum {\left(x_{i}-\bar{x}\right)}^{2}{\sum \left(y_{i}-\bar{y}\right)}^{2}}}$$

where r is the correlation coefficient we are interested in, $x_{i}$ is the mean decrease score of each predictor in a ML model, and $\bar{x}$ is the mean of the mean decrease scores of all predictors in the model. $y_{i}$ is the mean decrease score of each predictor in another ML model, and $\bar{y}$ is mean of the mean decrease scores of all predictors in another ML model. From the result in Table 5, strong positive correlations were observed between SMOTE_DT and SMOTE_XGB (0.86). Moderate-strong correlations were found between SMOTE_RF and SMOTE_XGB (0.68) and between SMOTE_XGB and SMOTE_RF (0.68). The top five predictors identified in SMOTE_RF, SMOTE_XGB, and SMOTE_DT were also significant in the SMOTE_LR model.

### 4.5. ML Model Performance of Nurse Turnover Prediction 

This study evaluated the performance of five different machine learning models using a confusion matrix. Table 6 summarizes the classification model indices, including TP, TN, FP, and FN. The validation dataset comprised 20% of the total data, with a sample size of 5295 individuals. Regarding TP, SMOTE_RF demonstrated the highest TP rate at 51.3%, correctly predicting the departure of 2714 out of 5295 individual nurses from their primary jobs. SMOTE_XGBT followed closely with a TP rate of 51.0%, accurately predicting 2701 departures. Specifically, SMOTE_RF correctly identified 2623 instances of nurses leaving their primary jobs, indicating that 51.3% of the cases predicted as job departures corresponded to actual departures. 

On the other hand, examining the FN, SMOTE_RF exhibited the lowest TN rate at 5.8%, predicting 312 out of 5295 cases as job departures when they did indeed leave their primary jobs. This implies that SMOTE_RF incorrectly classified instances as negative cases when they should have been positive. Thus, the model failed to identify only 312 cases in the positive class. Conversely, SMOTE_LR achieved the highest False Positive (FP) rate at 19.6%, correctly predicting 1039 out of 5295 nurses who did not leave their primary jobs. The model, however, missed 2450 instances that were part of the positive class. In terms of the proportion of correct predictions (TP+TN) in the confusion matrix, SMOTE_RF accurately classified 83.6% of the cases, SMOTE_XGBT achieved 82.7% accuracy, while SMOTE_DT and SMOTE_LR achieved 78.3% and 69.5% accuracy, respectively.

Table 7 evaluates five machine learning methods used in this study, using a set of commonly employed metrics for assessing machine learning algorithms. We have constructed classification metrics, specifically accuracy, recall (sensitivity), precision, and F1-score, to compare the performance of our models. Accuracy quantifies the number of correct classifications as a percentage of the total classifications made by a classification model. Precision represents the proportion of positive classifications that are accurately identified, while recall measures the proportion of all positive classifications correctly classified. The F1-score metric combines precision and recall using their harmonic mean. 

We employed a rigorous 10-fold cross-validation approach for the validation [30]. The dataset was divided through stratified random sampling, allocating 90% of the samples to the training set and the remaining 10% to the validation set. We ensured a non-overlapping representation of each class in both the training and validation sets.

After partitioning the training set into ten subsets, we applied the 10-fold cross-validation methodology to test and validate our models. According to the results obtained from the cross-validation analysis, SMOTE_RF demonstrated the highest accuracy among the evaluated models. This comprehensive validation process helped ensure the robustness and reliability of our model performance assessment.

In detail, when considering accuracy, SMOTE_RF and SMOTE_XGB emerged as the optimal models, each achieving similar accuracy scores of 74.39% and 73.88%, respectively. Conversely, SMOTE_LR (69.40%) and SMOTE_DT (69.90%) exhibited the lowest predictive accuracy. Examining precision, SMOTE_DT stands out as the best-performing model with a precision score of 83.77%. However, when evaluating the F1-score, SMOTE-XGB emerged as the optimal model at 71.45%, particularly when considering FN and FP to be of more significant concern.

On the other hand, considering the AUC, the model with the highest AUC score is SMOTE_RF, with an AUC of 77.67%. It is worth noting that AUC is not influenced by the threshold used in the ML classification or the distribution of the dataset. Thus, it provides a comprehensive measure of the classification power of the ML model. Consequently, SMOTE_RF is the preferred choice as the optimal model for predicting nurse turnover. It is interesting to note that our results are similar to the findings of Kim et al. [7]. In their study, RF was identified as the best predictive model. 

### 4.6. Optimized Random Forest Analysis Result

**Figure 6:**
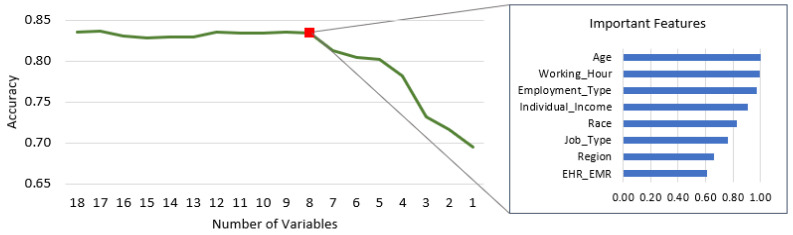
Optimal number of feature selections based on minimum accuracy.

In this section, we employed an optimized RF analysis to determine the optimal number of features based on their importance. We utilized 18 independent variables and 1 dependent variable for our model. The process involved running the model 18 times and progressively eliminating lower-scoring features. Our analysis revealed that the accuracy began to decline when only the top eight features in Figure 6 were retained. Consequently, we selected these eight features as the key predictors for the nurse turnover prediction problem. Age, Working Hours, Employment Type, Individual Income, Race, Job Type, Region, and EHR_EMR were the most important features of the recursive RF analysis. This dimensionality reduction enhances interpretability, especially for handling unbalanced characteristics, as demonstrated by [28]. Reducing the dataset’s dimensionality serves a valuable purpose. It equips the human resources department with a more accurate tool for predicting nurse turnover. Rather than concentrating on many predictive variables, the human resources department can achieve more effective interventions in reducing the turnover rate by focusing on smaller variables. Thus, the experimental findings offer valuable insights into reducing nurse turnover intention. In Table 8, we can see that SMOTE_RF shows better performance again for the index for accuracy, recall, precision, F1-score, and AUC than algorithms SMOTE_DT, SMOTE_XGB, and SMOTE_LR, which implies better predictive ability. 

## 5. Conclusions

The utilization of machine learning algorithms for processing raw employee turnover data represents a promising avenue for enhancing the capacity of human resource teams to address nurse turnover effectively. Through a comprehensive analysis of the key contributing factors to nurse turnover, it is possible to implement proactive measures aimed at its mitigation, facilitated by integrating machine learning algorithms.

The present study introduces an effective and efficient machine learning algorithm designed to predict nurse turnover utilizing the 2018 NSSRN dataset. The machine learning techniques proposed encompass LR, RF, DT, and XGB. To address the imbalanced datasets frequently encountered in the NSSRN dataset, we applied the SMOTE. None of the studies treated data imbalance problems of the NSSRN dataset when performing predictive analysis to predict nurse turnover. Our study demonstrates that by addressing the issue of imbalanced datasets through the SMOTE. This novel methodology effectively mitigates dataset imbalance in human resources, offering predictive insights that can empower healthcare managers and supervisors to take informed actions regarding factors influencing turnover intentions, thereby formulating intervention policies to retain their workforce. 

SMOTE_RF produced variable importance scores, which calculate the relative score of the different predictive factors. From the importance of predictor variable analysis, age, working hours, EHR/EMR usability, individual income, and household income were among the top five priorities in predicting turnover. We also used SMOTE_DT and SMOTE_XGB approaches to find the variable importance score, and a high correlation was observed among different models. Lastly, researchers used the SMOTE_LR approach to identify the significant predictive factors and to compare the result with SMOTE_RF. Five predictive factors found in SMOTE_RF were also substantial in the SMOTE_LR model. In summary, factors that reduce the likelihood of turnover include being in the NP category, residing in the South and West regions, using EHR or EMR technology, working standard hours, having high job satisfaction, ample job practice, being of white ethnicity, holding a BSN or MSN degree, and being young with a lower individual income.

### 5.1. Implications of the Study

This study’s results may interest healthcare managers or supervisors involved in staff management planning who wish to minimize the nurse turnover rate. The key considerations for practitioners include age, working hours, technology usability (EHR or EMR adoption), full-time versus part-time employment, geographic region, and job satisfaction. The literature consistently identifies these variables as influencers of turnover intentions. For instance, prior research by Cho et al. [20] noted a negative correlation between turnover intention and job dissatisfaction, while Blytt et al. [7] observed similar findings regarding overtime.

Our study found that the age variable emerged as the most significant factor in our SMOTE_RF analysis, with a notably high turnover probability observed among younger nurses. This observation is in alignment with the findings of several previous studies [6,20,31], all of which have highlighted age as a significant determinant influencing nurse turnover. The inclination for younger nurses to exhibit higher turnover rates can be attributed to various factors. New graduate nurses and those in the early stages of their careers often depart from their current positions to pursue better career prospects or improved employment benefits, such as higher income or more favorable job conditions [27]. Understanding that age plays a pivotal role in nurse turnover allows for us to consider it a potentially controllable factor within the healthcare sector. Proactive measures should be implemented by supervisors and managers to address this issue and mitigate the turnover intention among younger nurses [32]. These measures may include offering comprehensive job training, ample opportunities for on-the-job practice, and carefully assigning patients who require additional time to acclimate to their new work environment to new nurses. By taking such actions, healthcare institutions can better retain their younger nursing staff and ensure the continued delivery of high-quality patient care. This proactive approach acknowledges the significance of the age variable in nurse turnover and leverages it as a strategic point of intervention.

The second most crucial variable in our study is the “Working Hours,” specifically the impact of overtime on nurse turnover. Our findings underscore the substantial influence of overtime on the turnover rates among nurses, emphasizing the importance of addressing this issue. This insight can serve as compelling evidence to inform the development of optimal work scheduling practices and guidelines for nurse work scheduling aimed at minimizing nurse turnover, as advocated by Bae [7]. Overtime hours must be closely regulated to prevent nurse burnout, ensuring they can maintain their well-being and consistently deliver high-quality patient care. A key aspect of this regulation is continuously monitoring work hours and overtime. This monitoring should be a fundamental part of maintaining the quality of work within healthcare institutions [33]. It is particularly crucial during shift changes when uncertainties in hospital operations can result in unexpected overtime. Robust policies must be established during shift changes to address this challenge effectively, and supervisors or managers should actively advocate for implementing such changes. These measures are vital in maintaining a healthy work–life balance for nurses and ultimately contribute to reducing turnover rates and enhancing the overall quality of healthcare services. 

Our findings also underscore the strong association between nurses’ use of EHR or EMR technology and turnover intentions [34]. In the United States, the gray literature has reported higher job satisfaction among nurses using EHR systems. Nevertheless, issues such as poor EHR usability, the lack of standards, limited functionality, and the need for workarounds can detrimentally impact nurse productivity, patient care, and outcomes, as reported by Bjarnadottir et al. [34]. Adequate information and support are crucial to minimize potential harm caused by suboptimal EHR systems, as such improvements can enhance patient–nurse interactions and job performance, reduce medical errors, and alleviate nurse burnout and stress. Continuous support, financial incentives, and adherence to best practices should be integral components of the strategy to ensure the successful implementation of EHR or EMR systems in healthcare settings [35]. 

Finally, the nature of a nurse’s full-time or part-time employment significantly influences nurse turnover rates. Part-time nurses tend to exhibit a higher likelihood of turnover. This phenomenon can be explained by the practice of assigning part-time nurses to fill in for their full-time counterparts. Consequently, part-time nurses may find themselves less familiar with the routines, daily operations, and processes of the hospital wards or units, leading to apprehension about their work in the hospital setting. Implementing a buddy system could be an effective strategy to address this issue and mitigate the fear of work among part-time nurses [36]. This system would pair part-time nurses with more experienced and seasoned counterparts, providing them with the necessary support and guidance [5]. Such a support system can go a long way in helping part-time nurses acclimate to their work environment and foster a sense of confidence and belonging within the hospital [30]. Regardless of working environment, salary, region, and job satisfaction can also be considered to reduce nurse turnover. 

Our machine learning analysis has underscored the enhanced predictive power of SMOTE_RF when the number of variables is streamlined. This finding highlights the importance of prioritizing essential features and avoiding unnecessary information when addressing nurse turnover through interventions led by human resource teams, supervisors, or managers. Notably, SMOTE_RF consistently outperformed alternative methods across all performance metrics considered in this study.

### 5.2. Limitations of the Study

While our study yielded favorable results, there are still several limitations. The analysis primarily focused on the working environment and individual characteristics, mainly due to constraints imposed by the NSSRN dataset, which offered limited survey data results. Factors like leadership style, communication with management, individual health status, and collaboration with colleagues, which could significantly impact nurse turnover, were not incorporated into the model [3,11]. Future research should include these additional variables to ensure a more comprehensive analysis. Furthermore, researchers should explore alternative class imbalance methods beyond those employed in our study, as some of these approaches may offer more advanced and effective ways to examine nurse turnover. Researchers must also apply more sophisticated sampling techniques to address imbalances in predictive variables, a limitation in our current study. By addressing these limitations and adopting more comprehensive methodologies, we can further enhance our understanding of nurse turnover dynamics and contribute to developing more effective intervention strategies.

## Figures and Tables

**Table 1:** Description of feature used for ML analysis.

Feature Name	Data Type	Description
Turnover (Dependent Variable)	Categorical	Outcome feature: showing whether the nurse left the primary nursing position (1: Yes, 0: No)
Certificate	Categorical	Type of active certification (three-factor levels) NP: Nurse Practitioner, RN: Registered Nurse, Other: Combined variable (Clinical Nurse, Nurse Midwife, Nurse Anesthetist)
Region	Categorical	Location of primary nursing position-census division (four-factor levels: West, Midwest, South, and North)
Job_Satisfaction	Categorical	Levels of job satisfaction in primary nursing position (Satisfied vs. Dissatisfied)
Race	Categorical	Race (White vs. other race (Black or African American, Asian, American Indian or Alaska Native, Native Hawaiian or Other Pacific Islander, Other race))
Sex	Categorical	Sex (Male vs. Female)
Marital_Status	Categorical	Marital Status (Single vs. Married): widow, divorced, and separated is considered as Single
Veteran	Categorical	Veteran Status (Served vs. Never served): active duty for training and now or past active duty is considered as Served
Household_Income	Categorical	Pre-tax annual household income (three-factor levels): $75,000 or less, between $75,000 and $15,000, and more than $150,000
Degree	Categorical	Type of nursing degree: three-factor levels (AND: associate degree, BSN: Bachelor’s degree, MSN: Master’s degree, PhD/DNP/DN: Doctorate)
Dependent_6years	Categorical	A binary value indicating whether the nurse lives at home with a dependent who is less than 6 years old (Yes vs. No)
EHR_EMR	Categorical	Usability of Electronic Health Record (HER) or Electronic Medical Record (EMR) system (Yes vs. No)
Employment_Type	Categorical	Primary nursing position employment situation (Employed by the organization vs. other (employment agency as a traveling nurse, not as a travel nurse, and self-employed or working as needed))
Job_Type	Categorical	Full-time vs. Part-time work
Employment_Setting	Categorical	Type of work setting (three-factor levels: Hospital, Clinic/Ambulatory, and Inpatient + other work setting)
Practice	Categorical	Ability to practice to the extent of knowledge/education/training (Yes vs. No)
Working_Hour	Categorical	Number of hours worked in a typical week (Standard vs. Overtime); working hours greater than 40 is regarded as overtime.
Individual_Income	Numerical	Pre-tax annual earnings from primary nursing position ($)
Age	Numerical	Age of nurse

**Table 2:** Confusion matrix index.

Confusion Matrix		True Class	
		Positive (Turnorver = Yes)	Negative (Turnorver = No)
Predicted class	Positive (Turnorver = Yes)	TP (True Positive)	FP (False Positive)
	Negative (Turnorver = No)	FN (False Negative)	TN (True Negative)

**Table 3:** Distribution of the characteristics of the 18 extracted variables in the NSSRN database (original data and SMOTE).

Original Data					SMOTE			
Characteristic	Turnover		Turnover		Turnover		Turnover	
	Yes (N = 4728), 11%		No (N = 39,209), 89%		Yes (N = 11,349), 43%		No (N = 15,132), 57%	
Categorical Variables	Count	Percentage	Count	Percentage	Count	Percentage	Count	Percentage
Certificate								
Other	443	9.37%	2748	7.01%	1699	19.57%	1064	4.15%
NP	2173	45.96%	19,382	49.43%	4870	56.10%	7483	29.20%
RN	2112	44.67%	17,079	43.56%	2112	24.33%	17,079	66.65%
Region								
Midwest	1059	22.40%	8950	22.83%	2418	21.31%	3437	22.71%
North	893	18.89%	7227	18.43%	2548	22.45%	2706	17.88%
South	1574	33.29%	13,084	33.37%	3601	31.73%	5085	33.60%
West	1202	25.42%	9948	25.37%	2782	24.51%	3904	25.80%
Job_Satisfaction								
Dissatisfied	462	9.77%	3867	9.86%	2623	23.11%	1458	9.64%
Satisfied	4266	90.23%	35,342	90.14%	8726	76.89%	13,674	90.36%
Race								
Other Race	638	13.49%	5686	14.50%	2894	25.50%	2185	14.44%
White	4090	86.51%	33,523	85.50%	8455	74.50%	12,947	85.56%
Sex								
Female	4307	91.10%	35,847	91.43%	9128	80.43%	13,862	91.61%
Male	421	8.90%	3362	8.57%	2221	19.57%	1270	8.39%
Marital Status								
Married	3548	75.04%	29,490	75.21%	7514	66.21%	11,369	75.13%
Single	1180	24.96%	9719	24.79%	3835	33.79%	3763	24.87%
Veteran								
Never Served	4443	93.97%	36,919	94.16%	9599	84.58%	14,188	93.76%
Served	285	6.03%	2290	5.84%	1750	15.42%	944	6.24%
Household_Income								
Less than $75,000	1016	21.49%	8418	21.47%	2586	22.79%	3174	20.98%
$75,001 TO $150,000	2055	43.46%	17,369	44.30%	5090	44.85%	6706	44.32%
More than $150,001	1657	35.05%	13,422	34.23%	3673	32.36%	5252	34.71%
Degree								
ADN	773	16.35%	5891	15.02%	2240	19.74%	2215	14.64%
BSN	956	20.22%	9395	23.96%	2275	20.05%	3623	23.94%
MSN	2404	50.85%	20,308	51.79%	5025	44.28%	7873	52.03%
PHD/DNP/DN	595	12.58%	3615	9.22%	1809	15.94%	1421	9.39%
Dependant < 6 years								
No	3895	82.38%	32,248	82.25%	8970	79.04%	12,480	82.47%
Yes	833	17.62%	6961	17.75%	2379	20.96%	2652	17.53%
EHR_EMR Usability								
No	488	10.32%	4595	11.72%	2652	23.37%	1774	11.72%
Yes	4240	89.68%	34,614	88.28%	8697	76.63%	13,358	88.28%
Employment_Type								
Employed by Organization	4448	94.08%	36,540	93.19%	9259	81.58%	14,123	93.33%
Other	280	5.92%	2669	6.81%	2090	18.42%	1009	6.67%
Job_Type								
Full Time	3764	79.61%	30,964	78.97%	8106	71.42%	11,974	79.13%
Part Time	964	20.39%	8245	21.03%	3243	28.58%	3158	20.87%
Employment_Setting								
Clinical/Ambulatory	1608	34.01%	13,110	33.44%	3556	31.33%	5022	33.19%
Hospital	2058	43.53%	17,551	44.76%	4096	36.09%	6858	45.32%
Inpatient/Other	1062	22.46%	8548	21.80%	3697	32.58%	3252	21.49%
Practice								
No	1003	21.21%	8512	21.71%	4268	37.61%	3240	21.71%
Yes	3725	78.79%	30,697	78.29%	7081	62.39%	11,892	78.29%
Working Hour								
Standard	3197	67.62%	27,153	69.25%	7443	65.58%	10,552	69.73%
Overtime	1531	32.38%	12,056	30.75%	3906	34.42%	4580	30.27%
Numerical Variables	Average	Std.dev	Average	Std.dev	Average	Std.dev	Average	Std.dev
Age	55	11	48	12	50	11	49	12
Individual Income	70,285	41,404	85,444	37,157	80,471	41,404	84,069	37,157

**Table 4:** Predictors of nurse turnover using a SMOTE_LR algorithm.

Independent Variables	Odds Ratio	95% CI	*p*-Value
Certificate (ref: NP)			
Other	1.592	(1.42,1.78)	***
RN	1.032	(0.96,1.11)	
Region (ref: Midwest)			
North	1.037	(0.95,1.14)	
South	0.837	(0.77,0.91)	***
West	0.873	(0.80,0.95)	**
EHR/EMR Usability (ref: No)			
Yes	0.567	(0.52,0.62)	***
Employment Type (ref: Employed by Organization)			
Other	2.525	(2.29,2.78)	***
Job Type (ref: Full time)			
Part Time	1.446	(1.34,1.56)	***
Employment Setting (ref: Clinical/Ambulatory)			
Hospital	0.881	(0.82,0.95)	***
Inpatient/Other	1.248	(1.15,1.35)	***
Working Hour (ref: Overtime)			
Standard	0.732	(0.69,0.78)	***
Job Satisfaction (ref: Dissatisfied)			
Satisfied	0.469	(0.43,0.51)	***
Job Practice (ref: No)			
Yes	0.577	(0.54,0.62)	***
Race (ref: Other race)			
White	0.538	(0.50,0.58)	***
Sex (ref: Female)			
Male	2.111	(1.93,2.31)	***
Marital Status (ref: Married)			
Single	1.529	(1.43,1.64)	***
Veteran Status (ref: Never served)			
Served	2.154	(1.94,2.39)	***
Household Income (ref: $75,001 to $150,000)			
$75,000 or less	1.048	(0.96,1.14)	
More than $150,000	1.092	(1.02,1.17)	*
Degree (ref: ADN)			
BSN	0.726	(0.66,0.80)	***
MSN	0.730	(0.66,0.80)	***
PHD/DNP/DN	1.121	(1.00,1.26)	
Dependent less than 6 years old (ref: No)			
Yes	1.357	(1.25,1.47)	***
Individual Income	0.999	(1.00,1.00)	
Age	0.998	(0.99,1.01)	

* *p* < 0.05, ** *p* < 0.01, *** *p* < 0.001.

**Table 5:** Correlation of variable importance for three different models.

	SMOTE_RF	SMOTE_XGB	SMOTE_DT
SMOTE_RF	1		
SMOTE_XGB	0.683893	1	
SMOTE_DT	0.683749	0.861878	1

**Table 6:** Confusion matrix of five prediction models.

SMOTE_DT		True class	
		Positive	Negative
Predicted class	Positive	2623 (49.5%)	745 (14.1%)
	Negative	403 (7.6%)	1524 (28.8%)
SMOTE_XGB		True class	
		Positive	Negative
Predicted class	Positive	2749 (51.0%)	277 (6.1%)
	Negative	592 (11.2%)	1677 (31.7%)
SMOTE_RF		True class	
		Positive	Negative
Predicted class	Positive	2714 (51.3%)	561 (10.6%)
	Negative	312 (5.9%)	1708 (32.3%)
SMOTE_LR		True class	
		Positive	Negative
Predicted class	Positive	2450 (46.3%)	1039 (19.6%)
	Negative	576 (10.9%)	1230 (23.2%)

**Table 7:** The classification metrics for each machine-learning method.

Criterion	SMOTE_LR	SMOTE_RF	SMOTE_DT	SMOTE_XGB
Accuracy	69.40%	74.39%	69.90%	73.88%
Recall (sensitivity)	71.47%	82.12%	56.59%	83.77%
Precision	54.21%	60.33%	83.77%	62.28%
F1-score	61.65%	69.56%	67.55%	71.45%
Auc	69.50%	77.67%	73.97%	76.43%

**Table 8:** The classification metrics with eight feature selection.

Criterion	SMOTE_LR	SMOTE_RF	SMOTE_DT	SMOTE_XGB
Accuracy	70.39%	82.21%	74.84%	82.19%
Recall (sensitivity)	80.91%	90.52%	55.09%	81.12%
Precision	70.13%	82.36%	72.70%	89.72%
F1-score	75.05%	88.40%	62.62%	85.20%
Auc	73.24%	80.82%	76.29%	80.93%

## Data Availability

The Excel datasets generated and/or analyzed during the current study are available from the corresponding author upon reasonable request.
